# Regional Specific Evidence for Memory-Load Dependent Activity in the Dorsal Subiculum and the Lateral Entorhinal Cortex

**DOI:** 10.3389/fnsys.2017.00051

**Published:** 2017-07-25

**Authors:** Shih-pi Ku, Nozomu H. Nakamura, Nicolas Maingret, Liv Mahnke, Motoharu Yoshida, Magdalena M. Sauvage

**Affiliations:** ^1^Department of Functional Architecture of Memory, Leibniz-Institute for Neurobiology Magdeburg, Germany; ^2^Department of Physiology, Hyogo College of Medicine Nishinomiya, Japan; ^3^Mercator Research Group, Functional Architecture of Memory Unit, Ruhr-University Bochum, Germany; ^4^Faculty of Natural Science, Otto von Guericke University Magdeburg, Germany; ^5^German Center for Neurodegenerative Diseases (DZNE), Cognitive Neurophysiology Laboratory Magdeburg, Germany; ^6^Medical Faculty, Department of Functional Neuroplasticity, Otto von Guericke University Magdeburg, Germany; ^7^Center for Behavioral Brain Sciences, Otto von Guericke University Magdeburg, Germany

**Keywords:** subiculum, lateral entorhinal cortex, immediate early gene (IEG), *Arc* expression, recognition memory, proximal, deep LEC, superficial LEC

## Abstract

The subiculum and the lateral entorhinal cortex (LEC) are the main output areas of the hippocampus which contribute to spatial and non-spatial memory. The proximal part of the subiculum (bordering CA1) receives heavy projections from the perirhinal cortex and the distal part of CA1 (bordering the subiculum), both known for their ties to object recognition memory. However, the extent to which the proximal subiculum contributes to non-spatial memory is still unclear. Comparatively, the involvement of the LEC in non-spatial information processing is quite well known. However, very few studies have investigated its role within the frame of memory function. Thus, it is not known whether its contribution depends on memory load. In addition, the deep layers of the EC have been shown to be predictive of subsequent memory performance, but not its superficial layers. Hence, here we tested the extent to which the proximal part of the subiculum and the superficial and deep layers of the LEC contribute to non-spatial memory, and whether this contribution depends on the memory load of the task. To do so, we imaged brain activity at cellular resolution in these areas in rats performing a delayed nonmatch to sample task based on odors with two different memory loads (5 or 10 odors). This imaging technique is based on the detection of the RNA of the immediate-early gene *Arc*, which is especially tied to synaptic plasticity and behavioral demands, and is commonly used to map activity in the medial temporal lobe. We report for the first time that the proximal part of the subiculum is recruited in a memory-load dependent manner and the deep layers of the LEC engaged under high memory load conditions during the retrieval of non-spatial memory, thus shedding light on the specific networks contributing to non-spatial memory retrieval.

## Introduction

The most studied neuroanatomical pathway supporting spatial and non-spatial memory to date has been the trisynaptic loop. This pathway is thought to enable the transfer of information from the superficial layers (II and III) of the lateral and medial parts of the entorhinal cortex (the LEC and the MEC, respectively) to the dentate gyrus, from the dentate gyrus to the hippocampal subfield CA3, from CA3 to the hippocampal subfield CA1 and from CA1 to the deep layers (V and VI) of the LEC and MEC (Steward and Scoville, 1976; Swanson and Cowan, 1977; van Strien et al., 2009). However, the subiculum, consisting of several cortical fields located between CA1 and the EC, is the major output structure of the hippocampus (Amaral and Witter, 1989; Witter et al., 1989; Amaral et al., 1991; O’Mara et al., 2001). Moreover, this area has been suggested as the last stage of hippocampal processing to give rise to a complex episodic code that includes both spatial and non-spatial information, which is further directed to the neocortex (O’Mara, 2005). In comparison to that of the hippocampus, the function of the subiculum has been dramatically underexplored. The few available studies have mainly focused on its contribution to spatial information processing (Sharp, 1997, 2006; Anderson and O’Mara, 2004; Deadwyler and Hampson, 2004; Lever et al., 2009; Schon et al., 2016; Olson et al., 2017). However, the proximal part of the dorsal subiculum (close to CA1) receives extensive projections from the perirhinal cortex and the distal part of CA1 (bordering subiculum), which both play an important role in non-spatial recognition memory (Murray and Mishkin, 1998; Wan et al., 1999; Burke et al., 2011; Nakamura et al., 2013; Suzuki and Naya, 2014), suggesting a possible role of the subiculum in the mediation of non-spatial memory. In support to this hypothesis, two studies have reported the involvement of the subiculum in spontaneous object recognition memory and social transmission of food preference (Ross and Eichenbaum, 2006; Chang and Huerta, 2012). However, the extent to which the subiculum contributes to non-spatial memory is still unclear.

The LEC also receives some projections from distal CA1 and preferentially project to this area (Steward et al., 1976; van Groen et al., 2002, 2003). However, despite a well-accepted role in processing non-spatial information such as objects (Kesner et al., 2001; Deshmukh and Knierim, 2011; Deshmukh et al., 2012; Tsao et al., 2013; Reagh and Yassa, 2014), odors (Ferry et al., 2006; Xu and Wilson, 2012; Chapuis et al., 2013; Leitner et al., 2016) and texture (Boisselier et al., 2014), less than a handful of studies have specifically investigated its ties to non-spatial memory, especially associative memory (Tanninen et al., 2013; Boisselier et al., 2014; Igarashi et al., 2014). Thus, it is still unclear whether mediating non-spatial memory is a prominent role of the LEC. Importantly, since only the deep layers of the LEC have been shown to predict subsequent memory performance and not its superficial layers (Maass et al., 2014), we predicted that its engagement for the retrieval of non-spatial memory would be restricted to the deep layers.

In the present study we aimed at establishing whether the subiculum and the LEC play a critical role in non-spatial memory as well as reflect a memory load dependency. To assess whether the proximal part of the subiculum and the deep layers of the LEC contribute to the retrieval of non-spatial memory and whether this contribution depends on the memory load of the task, we studied patterns of neuronal activation during a delayed nonmatch to sample recognition memory task based on odors (DNMO; Nakamura et al., 2013). Because performing *in vivo* electrophysiology recording in specific cell layers is still a major challenge and using a lesion/inactivation approach was unlikely to yield the spatial resolution necessary to tease apart the involvement of the deep and the superficial layers of the LEC, a high-resolution molecular imaging technique (e.g., to the cellular level) was employed. This imaging technique is based on the detection of the expression of the immediate-early gene *Arc* which has been especially linked to plasticity processes and cognitive demands and has been recently used for mapping cognitive processes in the medial temporal lobe (Guzowski et al., 1999; Sauvage et al., 2013).

## Materials and Methods

### Subjects and Stimuli

Adult male Long-Evans rats (300–350 g; *n* = 5 DNMO animals with 10 odors and *n* = 4 with 5 odors, *n* = 4 controls) were maintained under reverse light/dark cycle (7 a.m. light off–7 p.m. light on) at a minimum of 90% of normal body weight, handled a week prior to the experiment and tested in their home cage. The stimulus odors were common household scents (thyme, paprika, coriander, etc.) mixed with playground sand. The scented sand was held in Nalgene plastic cups (one odor per cup), and a cup was fixed on a small platform lowered in the front part of the cage for testing. A pool of 40 household odors was available, 20 were used each day. This study was carried out in accordance with the recommendations and all procedures were approved by the Ruhr University Bochum Institutional Animal Use Committee and the LANUV (8.87-51.04.20.09.323).

### Behavioral Paradigm

**Figure 1:**
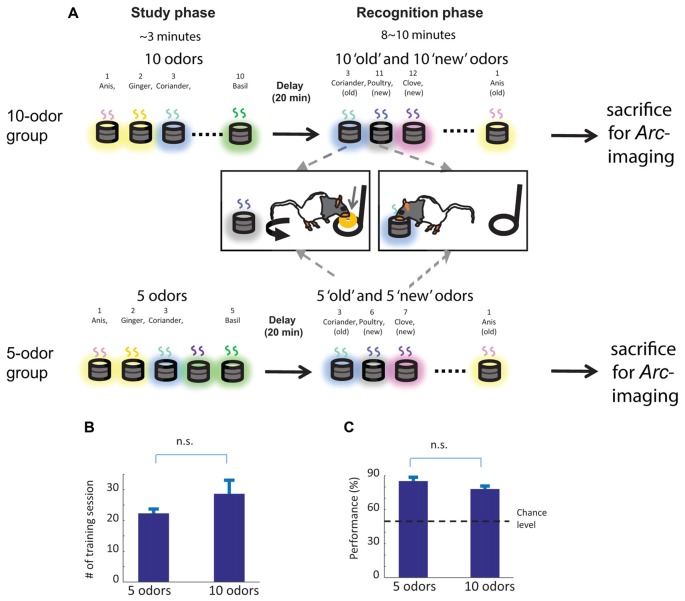
Delayed nonmatch to sample task based on odors, overview of the training scheme and memory performance on sacrifice day. **(A)** Top: experimental procedure for the 10-odor group. Ten odors were presented sequentially during the study phase. After 20 min delay, the memory for the 10 “old” studied odors was tested by presenting the “old” odors intermixed with 10 (“new”) odors. The testing odors were presented one at a time. To signal an “old” odor animals should repress digging into the test cup and go to the back of the cage to receive a cereal reward, if correct. To signal a “new” odor, animals should in the test cup to retrieve the same type of rewards. Bottom: experimental procedure for the 5-odor group: only the length of the study and the number of test stimuli changes compared to the 10-odor group. The figure is adapted and modified from Figure 1 in Nakamura et al. (2013). **(B)** The 5 and 10-odor groups learned to discriminate between “old” and “new” odors within a comparable number of sessions. **(C)** Memory performance on sacrifice day was also comparable between groups. n.s. not significant. Error bar: SEM.

Behavioral training followed the training protocol described in Nakamura et al. (2013). In brief, to study non-spatial recognition memory, we used the innate ability of rats to dig and to discriminate between odors. A pool of 40 odors was used. Odors were chosen in a pseudo random manner and care was taken that each odor was presented the same number of times to each animal. Each training session contained a “study” phase, a delay and a “recognition” phase (Figure 1A). Each day, rats were presented with a “study” list of 10 odors, which was different each day. During the recognition phase, animals were tested for their ability to distinguish between odors presented to them during the study phase (“old” odors) and additional odors (“new” odors) that were part of the pool of forty odors, but were not presented during the study phase (Figure 1A). To do so, animals were first trained to dig in the stimulus cups with unscented sand to retrieve one 1/4 of piece of Loop cereal (Kellogg’s Germany) after what they were trained on a delayed nonmatch to sample rule. During the recognition phase, when rats were presented with an odor that was part of the study list (“old” odor), rats were required to refrain digging, turn around and go to the back of the cage to receive a food reward: a correct response for an “old” odor (an “incorrect” response would be digging in the stimulus cup). Conversely, when the odor was not part of the study list (“new odor”), animals could retrieve a buried reward by digging in the test cup: a correct response for a “new” odor (an “incorrect” response would be going to the back of the cage to receive the reward). To ensure that the task could not be solved by smelling the reward buried in the sand, all cups were baited with a reward, but the reward was not accessible to the rats when an “old” odor was presented (e.g., trapped under a mesh). In addition, no spatial information useful to solve the task was available to the rats, given that testing cups for “new” and “old” odors were presented at the exact same location. Reward locations differed for the “new” and “old” odors (front and back of the cage, respectively), but were only experienced by the animals once a decision had been made (e.g., when the trial was over), hence could not contribute to behavioral performance. For both groups, training lasted approximately one and half months (Figure 1B, about 25 sessions, five sessions a week) and consisted of several steps during which the number of studied odors increased from one to five, the delay increased from 1 to 20 min, and the number of odors during the recognition phase increased from 2 to 10 (half “old”, half “new”). Animals transitioned between successive training stages when performance reached a minimum of 75% correct (Figure 1C) for two consecutive days. Once this criterion was reached for “5 odors study list” training stage (5 “study” odors, 5 min delay and 10 “test” odors), the group was divided into two groups of comparable accuracy. For the low memory load group, the “5 odors group” (*n* = 4), the delay between the study phase and the recognition phase was first extended to 10 min and subsequently to 20 min so that the final training stage of the 5 odor groups was as follows: 5 “study” odors, 20 min delay, 10 “test” odors. Animal belonging to this group received two training sessions per day so that animals experienced the same number of odors during the study and the test phase than the high memory load group (the “10 odors group”) over 1 day to avoid confounding the imaging results. For the high memory load group, the “10 odors group” (*n* = 5), first the number of study items increased to 10 odors, the delay to 10 min and the number of test stimuli to 20 odors. Subsequently, the delay was extended to 20 min so that the final training stage for this group consisted in 10 “study” odors, 20 min delay and 20 “test”odors. Once rats reached the criterion for the final stage of training (at least 75% correct over 2 days), animals were trained for one last day, sacrificed immediately after completion of the test phase and brain collected (all rats reached criterion on this day but one which reached 70%). All animals reached the final stage of training in approximately 27 testing days (about one and half month; see Figures 1B,C and “Results” Section). A group of control animals (*n* = 4), which was exposed to the same experimental conditions but did not perform the task, was used for measuring the baseline *Arc*-expression levels. Precisely, control animals were brought to the experimental room everyday together with rats performing the DNMO task. Every time when at least one DNMO rat reached the learning criterion, one control rat was taken direcly from their home cage and sacrificed together with the DNMO animal.

### Brain Collection

Animals were deeply anesthetized with isoflurane and decapitated. Brains were immediately collected, frozen in isopentane cooled in dry ice and subsequently stored at −80°C. Brains were then coronally sectioned on a cryostat (8 μm sections; Leica CM 3050S, Leica Microsystems, Wetzlar, Germany), collected on polylysine-coated slides and stored at −80°C.

### Detection of *Arc* Signal by *In Situ* Hybridization Histochemistry

The *Arc* DNA template was designed to amplify a fragment containing two intron sequences from bases 1934-2722 of the rat *Arc* gene (NCBI Reference Seq: NC_005106.2). DIG-labeled *Arc* RNA probes were synthesized with a mixture of digoxigenin-labeled UTP (DIG RNA Labeling Mix, Roche Diagnostics, Mannheim, Germany) and purified using Probe quant G-50 Micro columns (GE Healthcare). Fluorescent *in situ* hybridization histochemistry was performed as previously described (Nakamura et al., 2013). In brief, sections were fixed with 4% paraformaldehyde in sterile 0.1 M PBS. Sections were rinsed in PBS and acetylated with 0.25% acetic anhydride in 0.1 M triethanolamine-HCl. Following pre-hybridization incubation, hybridization solution (50% formamide, 5× standard saline citrate (SSC), 2.5× Denhardt’s solution, 250 μg/ml yeast tRNA, 500 μg/ml denatured salmon sperm DNA, and 0.05 ng/μl of digoxigenin-labeled *Arc* RNA probes) was applied to each slide (200 μl). The sections were coverslipped and incubated in a humidified environment at 65°C for 17 h. Sections were then rinsed in 5× SSC and then 0.2× SSC at 65°C for 1 h. Sections were incubated with 1% bovine serum albumin (BSA) in TBST buffer (0.1 M Tris–HCl pH 7.4, 0.15 M NaCl, 0.05% Tween 20) at room temperature, and then incubated with anti-digoxigenin-POD antisera (1/2000 dilution, Roche Diagnostics) in BSA/TBST at room temperature for 3 h. Sections were rinsed in TBST, signal amplified using the Tyramide Signal Amplification (TSA) Cy5 system (Cy5 plus system, PerkinElmer), counterstained with DAPI (1/100,000 dilution, Molecular Probe) and coverslipped. As controls for the staining, the detection was also performed without *Arc* probe, or with *Arc* sense probe, which led to the absence of *Arc* staining.

### Image Acquisition

**Figure 2:**
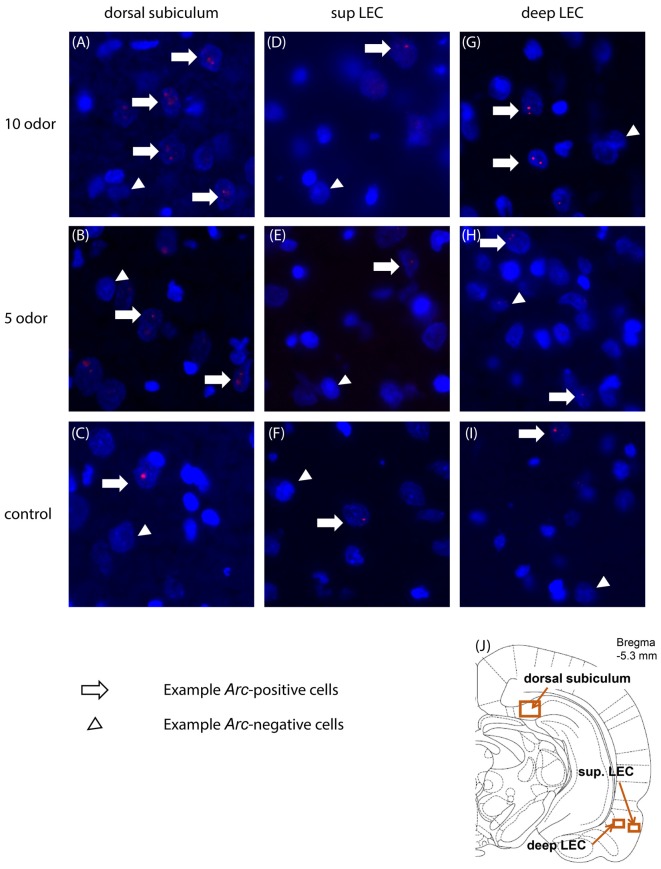
Representative images of *Arc* expression in dorsal subiculum **(A–C)**, superficial **(D–F)** and deep LEC **(G–I)** in the 10-, 5-odor and table control group. **(J)** Location of the imaging frames for the regions of interest. Sup, superficial; LEC, lateral entorhinal cortex.

CA1 and subiculum images were acquired using a 20× objective (Nikon) and LEC images were acquired using a 40× objective (Nikon) on a BZ-9000E fluorescent microscope (Keyence, Japan). A z-stack containing 12 images (subiculum: 545 by 724 μm; LEC: 272 by 362 μm; 0.7 μm thickness) was acquired per region of interest on at least three non-adjacent sections. The exposure time, contrast and gain settings were kept constant between image-stacks. Three regions of interest were chosen, according to the rat brain atlas (Paxinos and Watson, 2007; Figure 2J). Regions of interest were selected at the anteroposterior (AP) levels of −5.3 mm defined from the bregma. As featured in Figure 2J, for the proximal subiculum the right border of the image frame was placed adjacent to the CA1 pyramidal layer, and the frame was adjusted to cover the dorsal and ventral borders of the pyramidal layers of subiculum. For LEC, the top border of the frame was placed one frame below the border between the perirhinal cortex and the LEC based on cytoarchitecture of the region. For the superficial layers of the LEC the right border of the frame was placed in order that it included the entire layer II but not the layer I. For the deep layers of the LEC, the left border of the frame was placed adjacent to the dorsal endopiriform nucleus.

### Counting of *Arc* Positive Cells

The number of *Arc* pre-mRNA positive nuclei in the hippocampus was estimated as previously described (Nakamura et al., 2013). To account for stereological considerations, counting was performed on non-adjacent sections, and focused on the median 25% of each image-stack that was extracted from the z-stack and collapsed (West, 1999; Vazdarjanova and Guzowski, 2004). Briefly, non-neuron-like nuclei (~5 μm in diameter; the figure is adapted and modified from Figure 1 in Nakamura et al. (2013)) with intensely bright and uniform DAPI staining were excluded, whereas neuronal nuclei identified as large and diffusely DAPI stained were included in our analysis. *Arc* pre-mRNA positive nuclei were defined as cells carrying one or two signals with Cy5 tags within their nucleus, and the number of *Arc* pre-mRNA positive nuclei was counted on three frames per area of interest on non-adjacent coronal sections. Counting of *Arc* positive nuclei and *Arc* negative nuclei was performed manually by experimenters blind to experimental conditions with a software (ImageJ 1.46, National Institutes of Health, Bethesda, MD, USA) and the percentage of *Arc* positive cells per frame was calculated.

### Statistical Analysis

For the behavioral task, two-tailed *t*-tests were performed on the percent correct choice and the number of training sessions for comparing the 5-odor and 10-odor groups. One-sample *t*-tests were performed for comparisons to zero to study the baseline *Arc* expression for each area of interest. One-way and two-way ANOVA were carried out to compare proportions of *Arc* positive cells in the dorsal subiculum, superficial and deep layers of LEC and two-tailed *t*-tests as *post hoc* tests. All statistical analyses were performed using MATLAB (R2016b, Mathwork, Natick, MA, USA).

## Results

### Memory Performance and Number of Training Sessions Is Matched between Groups

The 5-odor and 10-odor groups learned to discriminate “old” from “new” odors within a comparable number of sessions (5-odor: 22.25 ± 1.09 sessions, 10-odor: 28.6 ± 3.91 sessions; *t*_(4)_ = 1.75, *p* = 0.16; Figure 1B) with a comparable accuracy (% correct: 5-odor group: 85% ± 3.33%; 10-odor group: 78% ± 2.85%; *t*_(4)_ = 1.82; *p* = 0.12; Figure 1C). Given that these two parameters do not differ between groups, the results reported in the present manuscript can neither be accounted by a between group difference in the number of training sessions nor by a group difference in memory strength.

### High Memory-Induced *Arc*-Expression in the Dorsal Subiculum

**Figure 3:**
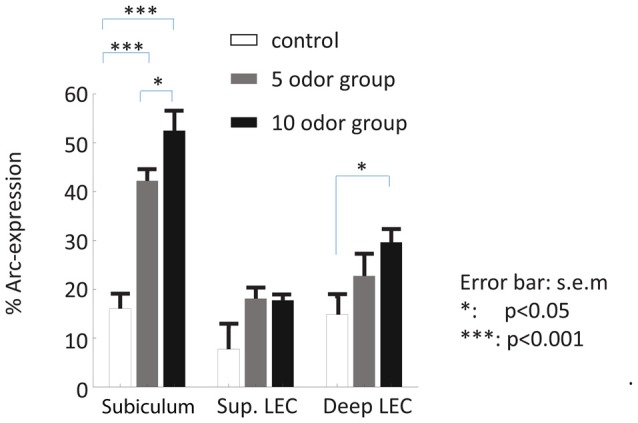
Patterns of activity in the subiculum and LEC of delayed nonmatch to sample recognition memory task based on odors (DNMO) rats and home caged controls. A high proportion of subicular neurons expressed *Arc* during the retrieval of non-spatial memory while comparatively a smaller proportion did in the superficial and deep layers of the LEC. The proportions of *Arc* positive cells varied in function of the memory load only in the subiculum and the deep layers of the LEC. *p < 0.05; ***p < 0.001; error bar: SEM.

We first examined whether the dorsal subiculum is recruited during the retrieval of non-spatial memories and whether this engagement depends on the memory load of the task (here: the presentation of 5 or 10 odors during the study phase). Statistical analyses suggest that it is the case as *Arc*-expression in this area was higher in the 5-odor and the 10-odor groups than in the control group (controls vs. 5-odors: *t*_(6)_ = 6.96; *p* = 0.00029; controls vs. 10-odors: *t*_(7)_ = 7.50, *p* = 0.00022; Figures 2A–C, 3) and the activity levels increased with the length of the study list (10 vs. 5-odors: *t*_(7)_ = 2.10, *p* = 0.0371). Thus, the results show that the dorsal subiculum is not only highly activated during non-spatial recognition memory retrieval, but that its recruitment dramatically is affected by the memory-load of tasks.

### *Arc* Expression Varies with the Memory Load of the Task in the Deep Layers of the LEC but Not in Its Superficial Layers

Here, we investigated whether the LEC, especially its deep layers, is recruited during the retrieval of non-spatial memory and the extent to which this engagement varies as a function of the memory load of the task. Since activity in the deep layers of the entorhinal cortex reflects subsequent memory performance but not that in the superficial layers (Maass et al., 2014), superficial and deep layers of the LEC were studied separately.

Statistical analysis of *Arc* expression in the deep layers between the three groups did not reveal a main “group” effect but showed that the activity level was higher in the 10-odor group than that of the controls (*F*_(2,12)_ = 4.47, *p* = 0.409; 10 odor group vs. controls: *t*_(5.7)_ = 3.33, *p* = 0.02; 10 vs. 5 odors: *t*_(7)_ = 1.4; *p* = 0.203; Figures 2G–I, 3). However, it was comparable between the low memory load group (5-odor group) and controls (*t*_(5.8)_ = 1.37 *p* = 0.22), suggesting a memory-load dependent recruitment of the deep layers of the LEC during the retrieval of non-spatial memories. In a striking contrast to the patterns of activity in the deep layers, levels of activation in the superficial layers of the LEC did not significantly differ between controls and both of the DNMO groups (5 or 10 odors, *F*_(2,12)_ = 4.34, *p* = 0.0441, 10 odors vs. controls: *t*_(3.2)_ = 2.11, *p* = 0.12; 5 odors vs. controls: *t*_(4.2)_ = 2.02, *p* = 0.11; 10 vs. 5 odors: *t*_(7)_ = 0.14, *p* = 0.889; Figures 2D–F, 3), indicating a lack of contribution of the superficial layers of the LEC to the retrieval of non-spatial memory. In summary, our results show that the deep layers of the LEC are selectively engaged in retrieving non-spatial information under high-memory load conditions while its superficial layers are not.

### The Dorsal Subiculum Is More Engaged during Non-Spatial Memory Retrieval than the Deep Layers of the LEC

Finally, because so few functional studies are available to directly compare the contribution of the dorsal subiculum and LEC to non-spatial recognition memory, we compared the extent to which the subiculum and the deep layers of the LEC were recruited at test. This analysis showed that the dorsal subiculum was strikingly more recruited than the LEC (an approximately two folds increase) for the retrieval of odor memory (main “area” effect: *F*_(1,25)_ = 20.61, *p* = 0.0098; main “group” effect: *F*_(2,25)_ = 20.46, *p* = 0.0004; interaction “area” by “group” effect: *F*_(2,25)_ = 5.62, *p* = 0.011; *post hoc*: sub vs. deep LEC: 10-odor: *t*_(8)_ = 4.69; *p* = 0.0016; 5 odor: *t*_(6)_ = 4.06; *p* = 0.0067), while baseline *Arc* expression was comparable between areas *t*_(6)_ = 0.259, *p* = 0.804). In addition, in further support to a predominant involvement of the dorsal subiculum and a higher sensitivity to memory load over the deep layers of the LEC, direct comparisons of activity levels between the high and low memory-load groups reached significance only in the subiculum as mentioned in previous paragraphs. Altogether, these last comparisons underline the possibility that the level of recruitment and the sensitivity to memory load is more robust in the dorsal subiculum than in the deep layers of the LEC, suggesting that the dorsal subiculum might play an even more prominent role than the deep LEC in the mediation of non-spatial memory.

## Discussion

In the present study we tested whether the dorsal subiculum and the LEC contribute to non-spatial recognition memory by imaging brain activity in rats performing a delayed nonmatch to sample task based on odors (DNMO). We bring evidence that, the proximal part of the dorsal subiculum was engaged during the recognition phase of the task in function of the memory load of the task. Besides, the deep layers of the LEC but not its superficial layers were also recruited under high memory load conditions. In addition, our results suggest that the dorsal subiculum might have an even more important role in non-spatial memory retrieval than the LEC.

### A Clear Memory-Load Dependent Recruitment of the Dorsal Subiculum during Non-Spatial Memory Retrieval

Here, we provide robust evidence that the dorsal subiculum is involved in processing non-spatial information and that its activity during the retrieval of memory varies dramatically with the memory load of the task. This finding of an involvement of the dorsal subiculum in the processing of non-spatial information is in line with the report of a role of this structure in the retrieval of social transmission of food preference, which preferentially relies on the ventral part of the medial temporal lobe (Countryman et al., 2005; Ross and Eichenbaum, 2006). It is also in agreement with another non-spatial study reporting that approximately 10% of the cells recorded with *in vivo* electrophysiology in the dorsal subiculum present novelty-detecting signals in an object recognition memory task (Chang and Huerta, 2012). A lesion of the subiculum did however not dramatically affect performance on this task in an independent study (Potvin et al., 2010). In addition, indirect evidence of a contribution of the subiculum to non-spatial memory has also emerged from spatial studies, for example memory deficits in a T-maze spatial alternation task caused by a dorsal subiculum lesion were found to be the clearest in the dark, suggesting a reliance of the subiculum on non-spatial (idiothetic) cues (Potvin et al., 2007). Even more critical, we show for the first time that this involvement depends on the memory load of the task, which demonstrates the role of the subiculum in mnemonic processes. Such memory-load dependency is also indirectly supported by a recent fMRI study in humans showing greater BOLD activity in the subiculum for higher memory loads during a delayed nonmatch to sample task, albeit during encoding and within the frame of a spatial working memory task (Schon et al., 2016). Characterizing the type process that the subiculum supports to mediate non-spatial memory is beyond the scope of this study.

Several hypotheses have been formulated to date. First, based on the bursting firing properties of a large-proportion of the subicular neurons (Anderson and O’Mara, 2003), e.g., the induction of bursts of action potentials in response to a single orthodromic stimulation, O’Mara (2006a) suggested that the subiculum might function to amplify hippocampal outputs. Thus the subiculum possibly contributes to reverse the inhibitory function of the dentate gyrus. The neurons in dentate gyrus fire infrequently and at low rates (Jung and McNaughton, 1993), which may act as a threshold for the hippocampal proper to filter the incoming information emerging from the entorhinal cortex (O’Mara, 2006a). This hypothesis is supported by the observation that *Arc*-expression in the subiculum of the DNMO rats appears to be about twice as high as that reported in the distal part of CA1 of the same animals (Nakamura et al., 2013). Of note, a direct comparison of *Arc* expression between the subiculum and CA1 requires for the control *Arc* expression for the subiculum reported here to be subtracted from that of the DNMO animals given that such normalization was performed in the study of Nakamura et al. (2013). Second, based on the pattern of anatomical input and output-projections, the subiculum has been suggested to support the integration between hippocampal mnemonic information and whole body movement related information (Staff et al., 2000; Martin, 2001; O’Mara, 2006b; O’Mara et al., 2009). Indeed, the subiculum receives major CA1 and cortical inputs from areas such as the entorhinal, perirhinal and prefrontal cortices, to which it returns prominent projections. In addition, it also receives inputs from and distributes to secondary cortices and subcortical areas (O’Mara, 2005). Taken together, findings in present study extend our knowledge on the contribution of the dorsal subiculum to non-spatial memory by bringing compelling evidence for a robust memory load dependent recruitment of this structure in memory retrieval.

### A Selective Engagement of the Deep Layers of the LEC during the Retrieval of Non-Spatial Memory

Our results also reveal a prominent role of LEC in non-spatial memory retrieval. This is in agreement with three recent studies which report that neuronal ensemble activity of the LEC coordinate with that CA1 during olfactory-spatial associative memory (Igarashi et al., 2014), that NMDA blockade impairs the acquisition of an olfactory-tactile associative learning (Boisselier et al., 2014) and that a lesion of the LEC impair memory expression in trace eye-blink conditioning (Tanninen et al., 2013). In contrast to the present study, these studies did focus on associative memory and not on single-item memory, and did not investigate whether LEC activity was memory-load dependent and whether functional laminar differences exist in the LEC. Our results indicate that the deep layers of the LEC are recruited during DNMO under the high memory load condition, bringing for the first time evidence for a laminar functional segregation in LEC within the frame of memory function and further supporting a critical role of the LEC for the retrieval of non-spatial memory. In line with these results, a high-resolution (7T) fMRI study in humans showed that subsequent memory performance depended on the activation of the deep layers of the entorhinal cortex during the encoding of novel scenes while processing novel information rather recruited its superficial layers (Maass et al., 2014). This study used however spatial stimuli and did not focus on the anterior-lateral part of the EC, the equivalent part of the rodent-LEC in humans (Maass et al., 2015; Navarro Schröder et al., 2015). Also, once normalized as it is the case in Nakamura et al. (2013), a direct comparison between *Arc* expression in distal CA1 in the same animals and that in the LEC reveals that the recruitment of the deep layers of the LEC is about only half as high as distal CA1. The technical approach adopted in this study does not enable us to dissociate whether these two patterns of activation reflect different or comparable computations. However, the role of the LEC and that of the hippocampus within the frame of recognition memory are believed to be qualitatively distinct, which could explain the difference in activity patterns. In summary, here we report that the deep layers of the LEC were recruited during non-spatial memory retrieval only under high memory demands, indicating that the LEC does contribute to non-spatial memory and that, within this specific frame, activity in the deep layers of the entorhinal cortex predicts subsequent memory performance.

### Higher Activity Levels in the Subiculum than in the LEC Might Suggest a Preponderant Role of the Subiculum in Non-Spatial Memory Retrieval When Compared to that of the LEC

Intriguingly, task-induced activity in the dorsal subiculum was drastically higher than in the deep layers of the LEC (approximately twice as high) despite a comparable baseline *Arc* expression in the two areas. In addition, the effect of the memory-load on *Arc* expression was also more robust in the subiculum than in the LEC: for the subiculum, the memory-load effect emerged also if a direct comparison between the 5 odor and the 10 odor groups is performed, while for the LEC the memory-load effect was illustrated by a significant difference between *Arc* expression in the 10 odor group and that of controls and a lack thereof between the 5 odor group and controls. Taken literally, these observations could be interpreted as reflecting a stronger involvement of the dorsal subiculum than the LEC in the retrieval of non-spatial memory. Importantly, the bursting firing properties of subicular neurons (Anderson and O’Mara, 2003) cannot account for the higher level of activity observed in the subiculum as such property could only explain an increase of activity within subicular cells and not an increase in number of activated subicular cells. Given the virtual lack of studies on the role of the subiculum in non-spatial memory as opposed to the healthy number of reports investigating the role of the LEC within this frame, it is challenging to drawn a firm conclusion on this point as further investigations would definitively be necessary to thoroughly test this hypothesis.

In conclusion, we brought here the first evidence of a memory-load dependent contribution of the proximal part of the subiculum and the deep layers of the LEC to non-spatial memory retrieval. In addition, our results tend to suggest a more prominent involvement of the subiculum than the LEC within this frame, which requires further investigations. Altogether, these results contribute to further characterizing the specific neuronal networks subserving non-spatial memory.

## Author Contributions

SPK analyzed the data and wrote the manuscript, NHN and LM performed the experiment and analyzed the data, NM performed the experiment, MY wrote the manuscript and MMS supervised the project and wrote the manuscript.

## Conflict of Interest Statement

The authors declare that the research was conducted in the absence of any commercial or financial relationships that could be construed as a potential conflict of interest.
